# Sleep apnea endotypes: from the physiological laboratory to scalable polysomnographic measures

**DOI:** 10.3389/frsle.2023.1188052

**Published:** 2023-05-24

**Authors:** Eysteinn Finnsson, Eydís Arnardóttir, Wan-Ju Cheng, Raichel M. Alex, Þóra B. Sigmarsdóttir, Snorri Helgason, Liang-Wen Hang, Jón S. Ágústsson, Andrew Wellman, Scott A. Sands

**Affiliations:** ^1^Nox Research, Nox Medical ehf, Reykjavík, Iceland; ^2^National Center for Geriatrics and Welfare Research, National Health Research Institutes, Miaoli, Taiwan; ^3^Department of Psychiatry, China Medical University Hospital, Taichung, Taiwan; ^4^Division of Sleep and Circadian Disorders, Department of Medicine, Brigham and Women's Hospital and Harvard Medical School, Boston, MA, United States; ^5^School of Nursing and Graduate Institute of Nursing, China Medical University, Taichung, Taiwan; ^6^Department of Pulmonary and Critical Care Medicine, Sleep Medicine Center, China Medical University Hospital, Taichung, Taiwan

**Keywords:** sleep disordered breathing, pathophysiology, precision medicine, endotyping, phenotyping

## Abstract

Obstructive sleep apnea (OSA) is a common disorder characterized by recurrent upper airway obstruction during sleep. Despite the availability of continuous positive airway pressure (CPAP) as the gold standard treatment, it is not well tolerated by all patients. Accordingly, research has increasingly focused on developing methods for OSA endotyping, which aims to identify underlying pathophysiological mechanisms of the disorder to help guide treatment for CPAP-intolerant individuals. Four key endotypic traits have been identified, namely: collapsibility, upper airway muscle compensation, arousal threshold and loop gain. However, most methods for extracting these traits require specialized training and equipment not available in a standard sleep clinic, which has hampered the ability to assess the full impact of these traits on OSA outcomes. This paper aims to provide an overview of current methods for OSA endotyping, focusing on the Endo-Phenotyping Using Polysomnography (PUP) method and its cloud-based extension, PUPpy, which offer scalable and accessible ways to estimate endotypic traits from standard polysomnography. We discuss the potential for these methods to facilitate precision medicine for OSA patients and the challenges that need to be addressed for their translation into clinical practice.

## Introduction

Obstructive sleep apnea (OSA) is a highly prevalent disorder that has major consequences for neurocognitive, cardiovascular, and metabolic health. Unfortunately, the leading therapeutic intervention, continuous positive airway pressure (CPAP), is limited by patient tolerance despite its otherwise excellent efficacy (Lozano et al., 2010; Weaver et al., 2012; Rotenberg et al., 2016; Bakker et al., 2019; Shapiro et al., 2021; NCA- CPAP, 2022; Šiarnik et al., 2022). Of the array of available and experimental non-CPAP interventions—including weight loss (Schwartz et al., 1991), oral appliances (Ng et al., 2003; Chan et al., 2010; Edwards et al., 2016a; Dissanayake et al., 2021; Pattipati et al., 2022), positional therapy (Randerath W. et al., 2021), hypoglossal nerve stimulation (Certal et al., 2015; Costantino et al., 2020; Op de Beeck et al., 2021b), pharyngeal surgery [particularly in pediatrics (Schwartz et al., 1992; Joosten et al., 2017; Gozal et al., 2020)], supplemental oxygen (Wellman et al., 2008), pharmacological interventions to: activate dilator muscles (e.g., atomoxetine-plus-oxybutynin) (Taranto-Montemurro et al., 2019, 2020; Hedner and Zou, 2022a; Schweitzer et al., 2022), decrease arousability from sleep (e.g., eszopiclone) (Eckert et al., 2011), and stabilize ventilatory control (carbonic anhydrase inhibitors) (Hedner and Zou, 2022a; Hedner et al., 2022)—each appears to be efficacious in some patients more than others. For the most part, non-CPAP therapies are administered in an empirical (i.e., trial-and-error) manner, with limited mechanistic information available to the clinician to predict the likelihood of a successful intervention in individual patients.

Over the last decade, the field of sleep medicine has come to the consensus that (1) there are different underlying pathophysiological causes of OSA (i.e., endotypic traits) (Younes, 2003; Younes et al., 2007; McGinley et al., 2008; Edwards et al., 2012, 2019; Sands et al., 2014), (2) that these traits differ considerably between patients (Wellman et al., 2011; Eckert et al., 2013; Xie et al., 2013; Sands et al., 2023), and (3) that individual differences in traits provide a mechanistic explanation for why some patients respond preferentially to one therapy over another (Wellman et al., 2008; Stanchina et al., 2015; Edwards et al., 2016a; Joosten et al., 2017; Landry et al., 2017; Sands et al., 2018a; Light et al., 2019). These concepts provide a potential avenue for *precision medicine*, whereby a subgroup of patients sharing a common underlying pathophysiology could be judiciously administered a therapy with preferential benefit. Accordingly, investigators have recently accelerated efforts to subclassify OSA based on mechanistic endotypic traits (i.e., endotypes) or other clinically-observable characteristics more generally (i.e., phenotypes), with the goal of better matching interventions to patients in a way that maximizes efficacy and tolerability (Edwards et al., 2019; Light et al., 2019; Malhotra et al., 2020).

### Key endotypic traits

There are at least four key endotypic traits that contribute to OSA (Younes et al., 2007; Ratnavadivel et al., 2010; Wellman et al., 2011; Eckert et al., 2013; Sands et al., 2018a; Light et al., 2019; Malhotra et al., 2020). Increased pharyngeal *collapsibility* is the primary determinant of OSA (Kirkness et al., 2008; Eckert et al., 2013; Sands et al., 2018b, 2023; Alex et al., 2022), and is characterized by an increased tendency of the pharyngeal tissues to obstruct the upper airway during sleep. Specifically, greater collapsibility manifests as a reduction in the ventilatory flow rate. Second, reduced pharyngeal dilator muscle activity is characterized by a failure of the dilator muscles (including the genioglossus muscle) to provide a normal baseline level of activation and/or the reflex increase in activation as ventilatory drive rises with obstruction (Wellman et al., 2011; Sands et al., 2018b). Low reflex *compensation* may be consequent to reduced neural responsiveness to stimuli and/or reduced neuromechanical efficiency. Third, a low *arousal threshold* is also a key trait contributing to OSA pathophysiology and is defined by a lower ventilatory drive threshold that triggers arousal (Heinzer et al., 2008; Wellman et al., 2011). Mechanistically, a lower arousal threshold places a limit on the ventilatory drive stimulus that the dilator muscles normally rely on to provide compensation support to the upper airway. Finally, a greater ventilatory instability or *loop gain* is defined as an excessive ventilatory drive response opposing a change in ventilation from baseline eupneic breathing (Wellman et al., 2011; Terrill et al., 2015). Despite being the hallmark of central sleep apnea, increased loop gain is also a key factor in the pathophysiology of OSA and is largely dominated by the dynamic ventilatory response to carbon dioxide (Younes et al., 2007). Conceptually, a higher loop gain contributes to OSA by exacerbating the transient loss of ventilatory drive stimuli needed to maintain muscle compensation in the presence of a vulnerable airway.

**Figure 1:**
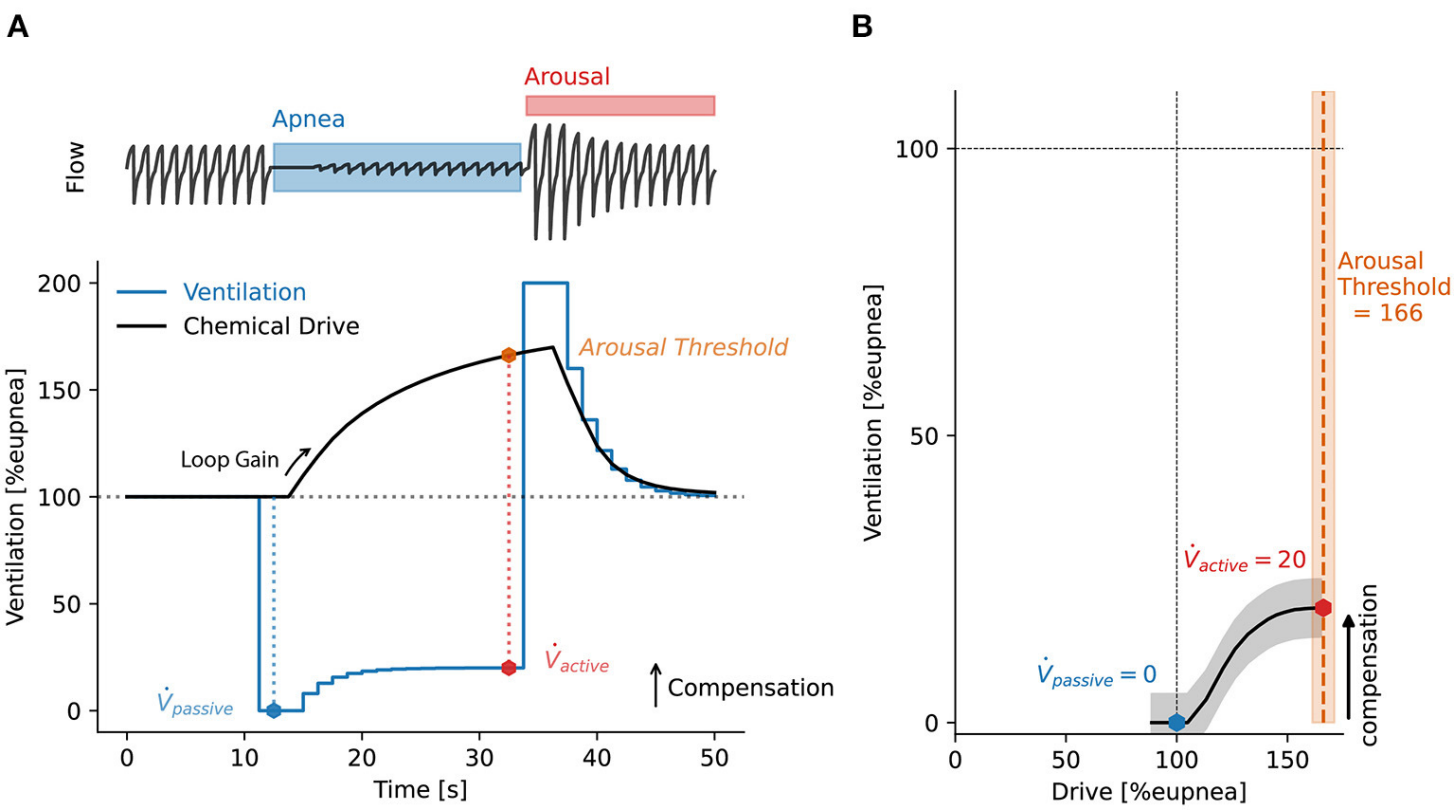
**(A)** Simulated respiratory event to illustrate the key concepts behind deriving sleep apnea endotypes from a ventilation signal. A simulated flow signal is shown above to help visualize the respiratory event. Ventilation is derived from the flow signal. A mathematical model of the chemoreflex control of breathing is fit to the unobstructed ventilation data, shown as a stepped blue line. The model is used to derive a continuous estimate of chemical drive, shown as a black continuous line. The chemical drive can be interpreted as the intended ventilation, which was not achieved due to the airway obstruction. Loop gain is then derived from the fitted model as the ratio of the output signal (chemical drive) to the input signal (ventilation). Collapsibility is measured as the ventilation at 100%_eupnea_ drive ($\dot{\text{V}}$_passive_). Arousal threshold is defined as the chemical drive preceding arousal. Upper airway muscle compensation ($\dot{\text{V}}$_comp_) is the difference in ventilation at eupneic drive (100%_eupnea_) and ventilation at the arousal threshold. **(B)** From the minute ventilation and drive data on **(A)** we create a ventilation-vs.-drive plot or “endogram.” Here, drive is binned into centiles and the median ventilation value within each drive bin is plotted against the median value of the binned drive data. The ventilatory endotypes: *compensation*, $\dot{\text{V}}$_passive_ and $\dot{\text{V}}$_active_ can be read directly from the plot. The endogram is used to aggregate and visualize the characteristic ventilation-drive relationships for a whole sleep study.

An important advance in the understanding of OSA pathophysiology is the notion that each of the four key traits are defined by ventilation and ventilatory drive (Younes, 2003; Wellman et al., 2011, 2013; Owens et al., 2015; Sands et al., 2018b) (see Figure 1). Collapsibility determines the ventilation during sleep at eupneic (normal resting baseline) ventilatory drive. Compensation is the increase in ventilation between eupneic drive and the maximum drive achievable during sleep, occurring at the arousal threshold, e.g. just before the termination of a respiratory event. Arousal threshold is the ventilatory drive that causes arousals. Loop gain is the ventilatory drive response to changes in ventilation from eupnea. Using this conceptual framework, it is possible to combine the endotypes mechanistically to explain the absence or presence of OSA and to understand the degree to which the traits causing OSA can be leveraged to ameliorate it (Wellman et al., 2013; Owens et al., 2015). For example, lowering loop gain is unlikely to be beneficial in patients with severe collapsibility and a poor muscle response, since such patients will incur pharyngeal collapse and loss of ventilation regardless of the level of ventilatory drive stimuli. For patients with ineffective upper airway muscles, raising the arousal threshold is unlikely to be helpful. These patients are expected to exhibit pharyngeal collapse regardless of their ability to tolerate increased ventilatory drive.

Accumulating evidence supports the concept that differences in these traits can explain responses to available and emerging CPAP and non-CPAP therapies. Key examples include the following: Patients with a high arousal threshold tend to adhere more to CPAP treatment, and increasing the arousal threshold pharmacologically with eszopiclone has been found to further improve CPAP adherence (Schmickl et al., 2020). Supplemental oxygen therapy to lower loop gain appears to be most efficacious in patients with less severe collapsibility, greater compensation, and higher loop gain (Wellman et al., 2008; Edwards et al., 2016b; Sands et al., 2018a). We caution that the use of hypnotics and supplemental oxygen as OSA therapies is still experimental and has not been approved for clinical use. Oral appliance therapy appears most beneficial in patients with less severe collapsibility and lower loop gain (Edwards et al., 2016a; Vena et al., 2020; Op de Beeck et al., 2021a), and may also be more efficacious in those with lower compensation and higher arousal threshold (Bamagoos et al., 2019). Hypoglossal nerve stimulation was most successful in patients with a higher arousal threshold, lower loop gain and good compensation; it may also be more efficacious in those with greater collapsibility (Op de Beeck et al., 2021b). On the other hand, according to a recent analysis (Wong et al., 2022), endotyping does not seem to be predictive of pharyngeal surgical outcomes. A key point emerging from the available response to therapy studies is that there is often no single trait that predicts the response to any non-CPAP therapy–even when the trait is explicitly targeted. Rather, knowledge of the traits in combination appears to be required. While further prospective validation studies are needed to confirm the use of endotypes in this context, knowledge of endotypic traits provides a promising means to identify subgroups of patients who are most likely to benefit from different therapies. Ultimately, clinically-applicable measurements of these traits will be needed before clinicians can utilize them to make treatment decisions for different subgroups of patients, i.e., precision medicine (Eastwood et al., 2011; Eckert et al., 2011; Edwards et al., 2012; Joosten et al., 2017; Randerath et al., 2018; Sands et al., 2018a; Bamagoos et al., 2019; Light et al., 2019; Taranto-Montemurro et al., 2019, 2020; Vena et al., 2020, 2022; Carter and Eckert, 2021; Op de Beeck et al., 2021a,b; Duong-Quy et al., 2022; Hedner and Zou, 2022b).

## Methods for quantifying endotypic traits

Here, we review current sleep apnea endotyping methodologies and how they can be translated from laboratory research into standard clinical practice. There are three streams of research methods for investigating endotypic traits. First, the simplest method for trait estimation is to relate direct output parameters from standard polysomnography (PSG) to the underlying sleep apnea pathophysiology [e.g., high apnea index (AI) as an indicator for high upper airway collapsibility]. These methods often require minimal additional calculations but do not take advantage of the wealth of mechanistic information available in PSG studies. Second, in the physiological laboratory, gold standard signals are *directly measured* to assess ventilation and ventilatory drive, with or without careful experimental procedures to manipulate ventilatory drive. Such studies typically seek to demonstrate physiological differences between patients or effects of therapies. These methods require invasive measurements using specialized equipment and training that are not available in standard sleep clinics. Finally, in the clinical setting, methods of *estimating* ventilation and ventilatory drive have been developed, with the goal of translating physiological knowledge from the physiology laboratory to the clinical arena where gold standard recordings are not feasible. Such studies use data collected during a routine sleep study and seek to provide a physiologically-sound means to predict the likelihood of responding to different interventions. These three approaches are summarized below.

### Pathophysiological insights from the polysomnography report

Useful but somewhat rough estimates of most OSA endotypes can be garnered without complex calculations or non-standard measurement equipment. One common approach is to estimate upper airway collapsibility from routine PSG indices as well as anthropometric measures. Several indicators have been explored in the literature, including the fraction of hypopnea vs. apnea (i.e., F_hypopnea_; lower values reflect greater collapsibility), apnea index (Vena et al., 2022), waist circumference, mean obstructive apnea duration, rapid eye movement apnea hypopnea index (REM-AHI), and non-REM obstructive apnea index (NREM-OAI) over NREM-AHI (Genta et al., 2020).

In addition, nadir oxygen saturation, high AHI and F_hypopnea_ have been found to be independent predictors of arousal threshold (Edwards et al., 2014a), and short respiratory event duration has been used as an indicator for increased arousability from sleep (Sands et al., 2018c; Butler et al., 2019). Quantifying loop gain from PSG using simple approaches has remained elusive. Algorithms have been developed for predicting high loop gain from the cyclical self-similarity of respiratory events during sleep (Oppersma et al., 2021). A simpler approach was proposed recently where higher AHI and lower hypopnea-percentage (i.e., F_hypopnea_) were used to predict higher loop gain values with moderate accuracy (Schmickl et al., 2022). Furthermore, high AHI during NREM vs. REM may indicate high loop gain, as loop gain has been shown to be lower in REM sleep (Landry et al., 2018; Joosten et al., 2021).

While, to our knowledge, no simple approaches have been published for deriving upper airway muscle compensation, there have been recent developments in training machine learning models to predict OSA endotypes and responses to treatment. These models utilize PSG variables and anthropometric measures as inputs and use machine learning or decision trees to classify patients for precision medicine in sleep apnea (Dutta et al., 2021, 2022).

Overall, these methods underline the fact that there is a wealth of physiologically relevant information in routine PSG reports that are not yet fully utilized for precision diagnoses.

### Specialized CPAP manipulation in the physiology laboratory

CPAP manipulations have been used for decades in OSA research to investigate OSA pathophysiology (Younes, 2003, 2004; McGinley et al., 2008; Wellman et al., 2011, 2013; Edwards et al., 2012, 2014b; Eckert et al., 2013; Sands et al., 2014; Messineo et al., 2018). Many permutations of these methods involved the following concepts: (1) Patients are placed on an optimal CPAP that resolves flow limitation and provides stable breathing. Conceptually, at quiet, stable breathing; ventilation and ventilatory drive are at eupneic levels and are considered to be equal to each other. (2) Abruptly “dropping” CPAP to a subtherapeutic level reveals a flow-limited airway with reduced capacity for ventilation due to the maximally “passive” pharyngeal dilator muscles. (3) Over time (e.g., with more gradual CPAP dial-downs), ventilatory drive rises and activates the pharyngeal muscles, which typically yields an improvement in ventilation that is considered to reflect dilator muscle compensation. (4) The arousal threshold is typically measured as the ventilatory drive (e.g., diaphragm EMG via catheter) or esophageal pressure on breaths preceding arousals during the experimental reductions in CPAP levels. (5) Measurement of gold standard loop gain typically involves quantifying the size of the increase in ventilatory drive that occurs in response to a controlled reduction in ventilation.

The most widely used approach for OSA endotyping avoids the need for invasive measurements of ventilatory drive through the use of judicious CPAP dial-ups to optimal pressure. The underlying basis for this method was that ventilatory drive equals ventilation during these periods (Wellman et al., 2011, 2013). This method allows for the derivation of the endotypes without the use of specialized equipment that is not present in a standard PSG lab, such as diaphragm EMG and esophageal/epiglottic manometry for measuring respiratory effort (Eckert et al., 2011, 2013; Sands et al., 2014; Edwards et al., 2016b). Using non-standard methodologies or equipment requires higher levels of training and longer setup time, and often results in a more invasive experience for the patient. These factors all hinder large scale adoption of the methods, despite their potential for guiding treatment selection (Terrill et al., 2015; Sands et al., 2018b; Finnsson et al., 2021).

Nonetheless, specialized CPAP manipulation studies require advanced training and are limited to only several laboratories worldwide. Specialized CPAP equipment that allows pressure drops to lower than 4 cmH_2_O are also not commercially available. Further, the average success rate of the CPAP drop method for estimating the four endotypic traits for each individual patient has been reported to be from 69% (Eckert et al., 2013) to 76% (Wellman et al., 2013), with difficulty initiating or maintaining sleep throughout the study procedures being a commonly reported issue (Edwards et al., 2012, 2014b; Eckert et al., 2013). The ratio of analyzable CPAP-drops per patient varied from a low of 16% (Edwards et al., 2014b) to a high of 70% (Eckert et al., 2013). These methods are therefore most suitable for assessing patients who are solid sleepers (higher arousal threshold) during periods of the night with the deepest sleep (Ratnavadivel et al., 2009, 2010). As a result, this approach is limited in its translational potential.

### Gold standard signals during spontaneous breathing without CPAP

An important step in the transition from the physiology laboratory to non-invasive clinical measurements involves the assessment of traits from spontaneous breathing, measuring ventilation and ventilatory drive, without the use of CPAP manipulation.

For many years, investigators have measured the arousal threshold without CPAP manipulation, using direct, invasive, measurement of the ventilatory drive (typically via catheters placed to assess negative esophageal or epiglottic pressures) prior to a scored arousal (Berry et al., 1996; Haba-Rubio et al., 2005; Eckert et al., 2011; Edwards et al., 2014a; Carter et al., 2016; Sands et al., 2018c). Extending this approach, a method was developed to assess collapsibility and muscle compensation directly from invasive measurement of ventilation (oronasal mask and pneumotach) and ventilatory drive (intraesophageal diaphragm EMG) (Sands et al., 2018b). The approach provides a ventilation-vs.-drive curve (see example in Figure 1B) describing pharyngeal mechanics that is conceptually similar to that measured via CPAP drops, but has the advantage of using direct measurement of these two variables, and captures the pathophysiology without disrupting the cyclic events that define the disorder.

### Polysomnographic method

**Figure 2:**
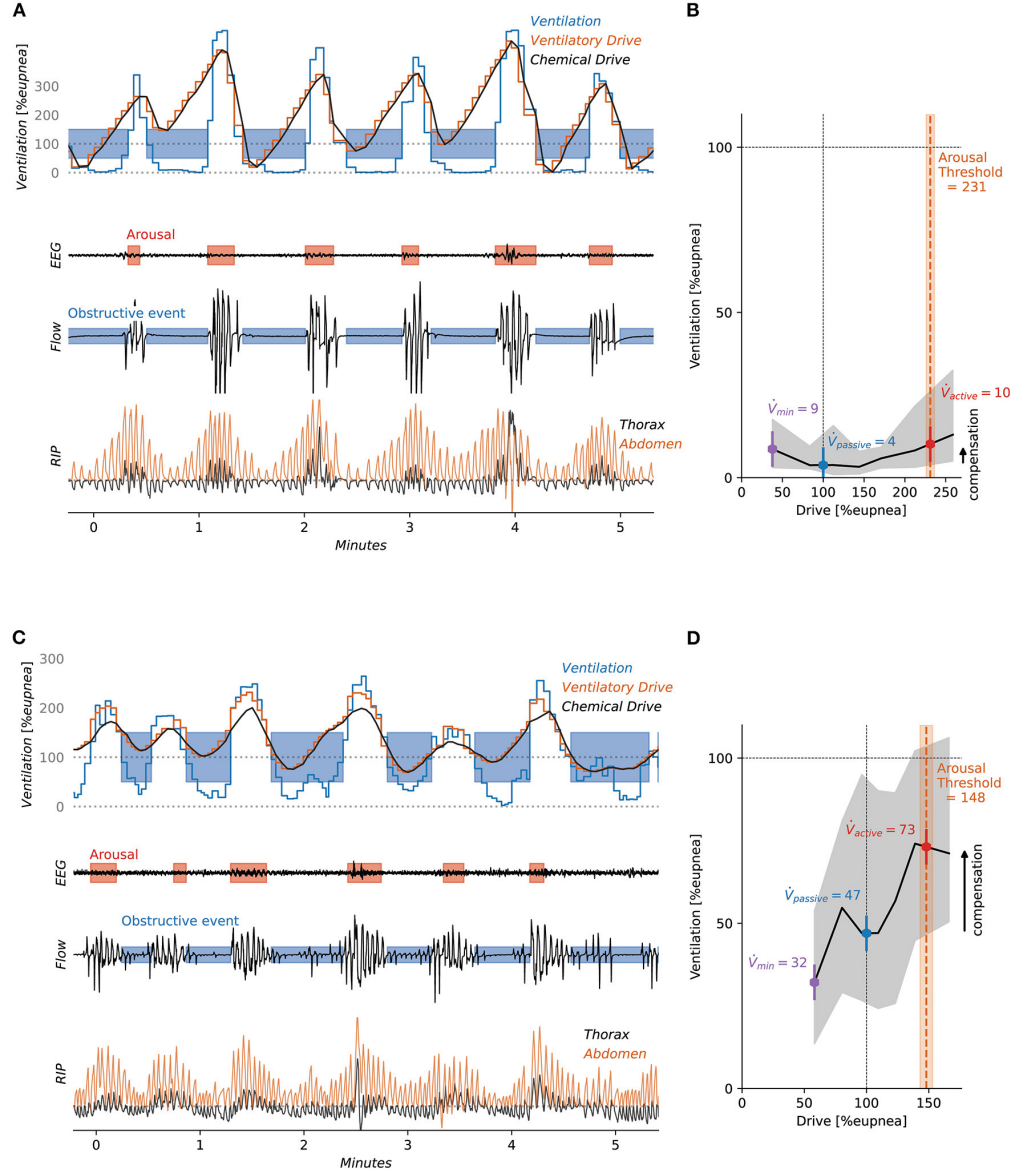
The figure shows two patients with different OSA expressions. Patient 1 **(A, B)** displays classic sleep apnea with high collapsibility and low upper airway compensation. Patient 2 **(C, D)**, has drive-dependent sleep apnea (Gell et al., 2022) where respiratory events correspond to reductions in chemical drive. As the drive increases the upper airway dilators are activated and some ventilation is restored, hence the upper airway compensation is larger.

The Endo-Phenotyping Using Polysomnography (PUP) method was developed by Sands and colleagues to translate the above methods to estimates that could be used clinically (Terrill et al., 2015; Sands et al., 2018b). The approach was designed to estimate the pathophysiological endotypes of OSA from a standard clinical PSG without the need for invasive measurements. Currently, this method extracts an estimate of ventilatory flow from the nasal pressure signal. Tidal volume is calculated by integrating the flow signal, where ventilation is derived by dividing the tidal volume by each breath's duration. Ventilation is presented as a percentage of a local 7-min average, with 100% considered to represent eupneic ventilation. Thus, ventilation at 0% represents a complete apnea, 100% is eupneic breathing, and >100% is hyperpnea. Rather than invasively measuring ventilatory drive, an estimate is calculated from the ventilation signal, leveraging the assumption that ventilation reveals the ventilatory drive when the airway is open but not when it is obstructed. A drive estimate is derived using a chemoreflex model which takes the ventilation signal as input and outputs chemical drive according to the dynamics dictated by the model parameters (gain, time constant, delay). This chemical drive signal is best fit to the ventilation signal using least squares; specifically, the chemoreflex model parameters are adjusted. In addition, the presence of arousal is also used in the model. Namely, an additional wakefulness/arousal drive is considered during any breath that lies within the margins of a scored arousal; a single additional parameter (ventilatory response to arousal) (Edwards et al., 2013) is added to the chemical drive to yield the overall ventilatory drive; when arousals are scored, it is this ventilatory drive signal that is best fit to the ventilation signal. Goodness of fit, for least squares minimization, is only evaluated between scored events, i.e., when the airway is expected to be unobstructed. These estimates of ventilation and ventilatory drive are used in place of the gold standard signals (Terrill et al., 2015; Sands et al., 2018c; Finnsson et al., 2021; Gell et al., 2022). An illustration of estimated ventilation and ventilatory drive is shown in Figure 1A. With the normalized ventilation values and corresponding drive values, the PUP method can be used to derive loop gain (Terrill et al., 2015), arousal threshold (Sands et al., 2018c), upper airway collapsibility, and upper airway compensation (Sands et al., 2018b). Typically, these traits are derived and presented for NREM sleep. See an application of the method to patient data in Figure 2.

#### Scalability

PSG endotyping has since been used in multiple research applications (Wellman et al., 2011, 2013; Terrill et al., 2015; Sands et al., 2018b,c; Taranto-Montemurro et al., 2019, 2020; Finnsson et al., 2021; Alex et al., 2022), primarily in studies seeking to identify a patient subgroup that responds preferentially to existing and experimental interventions. However, authors of the work have required specialized software (MATLAB) and some training to independently generate trait data. To demonstrate that the approach is truly *scalable*, our team recently introduced “PUPpy” (Finnsson et al., 2021), a new independent implementation in the Python programming language of the PUP method, that was originally implemented in MATLAB. PUPpy is a cloud-enabled solution based on the original PUP method principles (Finnsson et al., 2021), and directly provides the user with endotypic trait values from uploaded clinically-collected PSG data. The trait values are congruent with the PUP method and demonstrated that there are no major hurdles anymore to making the analysis widely accessible to researchers and clinicians. To maintain the alignment of the PUPpy method with the original validation of the PUP method, it would be helpful to validate it against gold standard methods (e.g., CPAP drop method/gold standard ventilation and drive signals) to provide an opportunity for ongoing enhancement and development.

### Normative values and demographic differences

As endotyping is an emerging field in sleep research, thresholds for abnormal endotypic trait values have not yet been established. Several studies have reported the range of values of different endotypic traits calculated using the PUP method. Table 1 describes two previously published datasets where PUP has been used for analysis: Osteoporotic Fractures in Men Study (MrOS) and Multi-Ethnic Study of Atherosclerosis (MESA) (Blackwell et al., 2011; Chen et al., 2015; Zhang et al., 2018; Alex et al., 2022). Here we also include a new dataset collected in Taiwan at the China Medical University Hospital (CMUH) that is unique for its clinical population, which we analyzed using PUPpy (Finnsson et al., 2021). The low, moderate, and high values for each trait are described for each of the three datasets based on their tertiles (Table 2). Notably, compared to the community studies, the clinical population appears to exhibit greater collapsibility and lower compensation, consistent with greater pharyngeal deficits, as expected. The clinical population also exhibited a higher average arousal threshold, perhaps a reflection of an increased physiological “sleepiness” (Edwards et al., 2014a) that may be expected of symptomatic individuals attending a sleep clinic. It is also possible that differences in race/ethnicity of the populations contribute to these differences, noting that Asian populations often exhibit greater anatomical compromise and collapsibility in obesity-adjusted analyses (O'Driscoll et al., 2019). Further understanding of the OSA etiology of different demographics via sleep apnea endotyping has the potential to provide insight into optimal treatment pathways.

**Table 1:** Descriptive statistics and endotype values in NREM sleep for different cohorts.

	**PUP with cannula flow and modeled drive in NREM sleep**	**PUP with cannula flow and modeled drive in NREM sleep**	**PUPpy with cannula flow and modeled drive in NREM sleep**.
	***N*** = **2,316 [MrOS]**	***N*** = **1,792 [MESA]**	***N*** = **765 [CMUH]**
Setting	Unattended in-home PSG, manually scored	Unattended in-home PSG, manually scored	In-lab PSG, manually scored PSG
Population	Community cohort	Community cohort	Clinical cohort
Age	76 [72–80]	68 [61–76]	41 [34–51]
Sex (M:F)	2316:0	866:926	641:124
Race/Ethnicity	3% Black, 91% White, 2% Hispanic, 3% Asian, 1% Other	27% Black, 24% Hispanic, 12% Chinese, 37% White	100% Southeast Asian^***^
BMI	27.0 [25.0–30.0]	28.2 [25.0–32.1]	28.9 [25.8–32.4]
AHI	20.0 [12.0–33.0]	20.8 [12.2–34.9]	25.9 [13.3–52.1]
Collapsibility ($\dot{\text{V}}$passive, %)^†^	71.5 ± 15.6	77.5 ± 14.4	62.0 ± 22.8
Collapsibility ($\dot{\text{V}}$min, %)	50.9 ± 22.5	63.7 ± 20.4	50.5 ± 17.2
Compensation ($\dot{\text{V}}$active-$\dot{\text{V}}$passive, %)	5.7 ± 24.1	6.1 ± 18.2	−3.6 ± 20.3
Loop gain (${\text{LG}}_{1}^{*}$)	0.62 ± 0.17	0.58 ± 0.18	0.55 ± 0.18
Loop gain (LGn^**^)	0.50 ± 0.12	0.44 ± 0.11	0.38 ± 0.10
Arousal Threshold (%)^†^	148.2 ± 26.5	139.5 ± 23.9	159.10 ± 28.26

Values either presented as Median [IQR] or Mean ± SD ^*^LG_1_, the loop gain at 1 cycle/min, is typically used for endotyping from PSG instead of the steady-state loop gain (LG_0_). This is because natural respiratory events rarely reach steady state, so LG_0_ does not have good observability. LG_1_ is more accurately observed since 1 cycle/min is closer to the frequency of respiratory events. ^**^LGn represents the loop gain at the system's natural frequency. ^***^Based on knowledge of the Taiwanese population. ^†^The values for Collapsibility ($\dot{\text{V}}$passive) and Arousal Threshold have been square root transformed for normality (Sands et al., 2018a; Alex et al., 2022 - Identifying obstructive sleep apnoea patients responsive).

**Table 2:** 33^rd^ percentile and 66^th^ percentile (i.e., trentiles) of the endotypic traits in NREM sleep for different cohorts.

	**33.3 percentile**			**66.6 percentile**		
	**MrOs**	**MESA**	**CMUH**	**MrOs**	**MESA**	**CMUH**
Collapsibility ($\dot{\text{V}}$passive, %)^†^	70.3	76.8	58.7	78.7	83.7	75.5
Collapsibility ($\dot{\text{V}}$min, %)	46.1	63.2	45.7	64.9	74.8	59.4
Compensation ($\dot{\text{V}}$active-$\dot{\text{V}}$passive; %)	4.1	4.3	−7.4	11.1	8.2	6.2
Loop gain (LG1)	0.54	0.49	0.46	0.68	0.62	0.60
Loop gain (LGn)	0.45	0.38	0.34	0.55	0.47	0.42
Arousal threshold (%)^†^	134.9	127.7	142.4	156.0	144.4	165.3

^†^The values for Collapsibility ($\dot{\text{V}}$passive) and Arousal Threshold have been square root transformed for normality (Sands et al., 2018a; Alex et al., 2022 - Identifying obstructive sleep apnoea patients responsive).

## Future methodological developments

The non-invasive PSG method described has several limitations with respect to accurately capturing ventilation and ventilatory drive. A recent debate on the topic highlighted some of the limitations of the method as well as giving suggestions for improvements (Sands and Edwards, 2023; Younes and Schwab, 2023). Here, we discuss areas of current development.

### Limitations of manual scoring of respiratory obstruction

A fundamental assumption of the model-based endotype approach is that the airway is unobstructed and not flow-limited during recovery hyperpnea as well as during periods where no respiratory events are scored (Terrill et al., 2015; Sands et al., 2018b; Finnsson et al., 2021). Recently our colleagues showed that some patients consistently exhibit flow-limited recovery breaths (Mann et al., 2021). In those patients, the ventilatory drive will be underestimated. To mitigate this, continuous measures of the severity of upper airway obstruction could be used to improve the model-estimated drive signal. Flow-shape-derived (Mann et al., 2019, 2021; Parekh et al., 2021) and RIP-derived (Finnsson, 2017; Parekh et al., 2021) breath-level obstruction measures have been explored with promising results. Continuous quantification of obstruction could further enhance the precision of drive and ventilation estimates in the presence of sustained flow limitation and a concomitant rise in baseline drive above eupneic levels.

### Passive upper airway collapsibility

Passive upper airway collapsibility is most commonly represented by P_crit_ (Kirkness et al., 2008; Eckert et al., 2013) and represents the x-intercept of a plot of airflow or ventilation vs. CPAP pressure level. P_crit_ can be interpreted as the theoretical CPAP pressure level where the airway closes. Although P_crit_ has been considered a gold standard method for upper airway collapsibility, it requires manipulation of airway pressures (Kazemeini et al., 2022) and is inherently not observable during spontaneous breathing. By contrast, the y-intercept of the same ventilation-vs.-CPAP relationship, called “$\dot{\text{V}}$_passive_,” similarly provides a gold standard collapsibility measure in units of ventilation (Younes, 2003). $\dot{\text{V}}$_passive_ represents the maximum level of ventilation that can be achieved at normal ventilatory drive through a passive airway at atmospheric pressure. Importantly, patients spend their time spontaneous breathing at atmospheric pressure (by definition), so this variable is potentially both observable and physiologically relevant to their OSA pathophysiology.

Estimating passive upper airway collapsibility (ventilation at eupneic drive, “$\dot{\text{V}}$_passive_”) from non-invasive signals during spontaneous breathing requires an accurate assessment of the ventilatory drive. As discussed above, any underestimation of the ventilatory drive is expected to provide an overestimation of $\dot{\text{V}}$_passive_. To address this concern, Vena et al. recently examined an alternative measure of passive collapsibility: ventilation at nadir drive ($\dot{\text{V}}$_min_), a measure which is independent of systematic bias in drive levels (Vena et al., 2022). P_crit_ was found to be more strongly correlated with $\dot{\text{V}}$_min_ (r = −0.54) than it is with $\dot{\text{V}}$_passive_ (r = −0.38). We emphasize, however, that a perfect correlation is not necessarily expected since P_crit_ and $\dot{\text{V}}$_min_/$\dot{\text{V}}$_passive_ are inherently different measures. Nonetheless the modest correlation indicates that there remains room for further development. We also emphasize that $\dot{\text{V}}$_passive_ measured from spontaneous breathing is systematically greater than that measured following an acute reduction in CPAP, likely because the baseline dilator muscle activity is greater off CPAP than immediately after an abrupt CPAP drop, even at similar drives. While the spontaneous breathing methods capture less of a truly passive tissue mechanical behavior, it may be advantageous to quantify the degree of collapsibility as it contributes to the pattern of cyclic events that define each patient's disorder.

### Oral breathing

A key limitation of nasal pressure is that it captures nasal rather than combined oronasal airflow. Unfortunately, oral breathing is both prevalent and significant in OSA (Gleeson et al., 1986; Nascimento et al., 2019) and can be invoked by obstructive respiratory events (Suzuki et al., 2015; Lebret et al., 2018). Errors in trait estimates are expected for those with the most pervasive mouth opening during sleep (Redline et al., 2007).

Several auxiliary flow sensors have been proposed to mitigate the effects of oral breathing. An oronasal thermistor is frequently invoked (Redline et al., 2007), yet this sensor technology does not provide a linear flow measurement for quantitative use (Farré et al., 1998; Redline et al., 2007). When properly calibrated and processed, respiratory inductance plethysmography (RIP) can be used to assess ventilation (Montazeri et al., 2021). With careful calibration, RIP could provide a flow signal to generate traits similar to those derived from oronasal flow and may provide a more reliable alternative to nasal pressure in circumstances with considerable mouth breathing.

### Repeatability and physiological variability

It is established that some traits vary within a night. Given both physiological variability and measurement noise, there may be concerns about repeatability of estimated trait values generated from PSG methods. Most notably, collapsibility is greater in supine position than in lateral position (Ong et al., 2011) but often appears unaffected by sleep stage (Ong et al., 2011; Joosten et al., 2021; Messineo et al., 2022). Loop gain, however, is lower during REM sleep than during NREM (Joosten et al., 2021). Interestingly, upper airway muscle compensation is largely unaffected by state (Messineo et al., 2022). As with prior physiology studies, trait values reported by the PUP method for these traits are the medians during NREM for the night of study, and physiological variations are incorporated into the 95 percentile confidence intervals of the estimated values.

The traits derived from PUP have been shown to have a moderate-to-good within-night repeatability, with correlations (Pearson correlation) ranging from 0.69 to 0.83 (Alex et al., 2022) for two independent measures taken from the same night. Night-to-night repeatability is similar, with correlations (intra-class correlation) ranging from 0.72 to 0.83 (Strassberger et al., 2023) in one study, and 0.67–0.91 in another (Tolbert et al., 2023). In general, intraclass correlations for collapsibility, loop gain, and arousal threshold have been >0.8 but lower for compensation. Of note, compensation is calculated as the difference in two collapsibility measures ($\dot{\text{V}}$active minus $\dot{\text{V}}$passive) such that measurement error is augmented. Overall, however, night-to-night repeatability is similar to that observed for apnea-hypopnea index (Alex et al., 2022). Long term repeatability (6–7 years between studies), at least in an elderly male population, has been shown to be more modest with r = 0.36–0.61.

With the goal of increasing repeatability, incorporating the effects of sleep state, position, arousal intensity (Azarbarzin et al., 2014; Amatoury et al., 2016), or other covariates may be beneficial, but the optimal means to do so remains an area for future research.

## Future clinical utility

Over the last decade, the field of sleep medicine has actively investigated novel clinically-applicable measurements that capture differences in underlying disease pathophysiology to aid clinicians in selecting the most appropriate treatments for their patients (Wellman et al., 2011; Carberry et al., 2018; Schmickl et al., 2018; Sutherland et al., 2018; Edwards et al., 2019; Light et al., 2019; Martinez-Garcia et al., 2019; Lyons et al., 2020; Siriwardhana et al., 2020). Until now, clinical interventions for the treatment of OSA have primarily followed a one-dimensional treatment pathway: A single diagnostic parameter, the AHI, or one of its analogs (Epstein et al., 2009; LCD, 2022; NCA- CPAP, 2022) was used to determine the need for a single intervention, CPAP. This pathway from diagnosis to treatment involves minimal mechanistic information which may lead to simplistic decision-making, which in turn may deter new physicians from entering the field (Watson et al., 2017). On the other hand, OSA is a highly complex disorder that manifests as the downstream product of interacting deficits in multiple pathophysiological traits, and involves aspects of upper airway anatomy, pulmonary mechanics, ventilatory control, pharyngeal muscle control, and sleep neurobiology. Unfortunately, many of these academically challenging aspects are not currently considered in daily clinical sleep medicine to benefit patients. Novel clinically available tools to capture these underlying mechanisms could potentially change the nature of clinical sleep medicine. Once available–and supported by data on their clinical utility–we consider that clinicians will have the means available to better understand the etiology of an individual's disorder and use this information to select the optimal treatment for their patient.

The pathway to widespread use of endotypic traits is expected to involve several research and clinical challenges. Greater accuracy of the estimates of traits through improved processing and estimation of ventilation and ventilatory drive will be needed. Such studies will need to be guided by gold standard signals obtained from physiology laboratory settings with simultaneous clinical signals. The field will further benefit from a shift toward improved respiratory signal fidelity (perhaps in the opposite direction to the current approach of determining how few signals can be used to obtain a diagnostic AHI value). Currently, some in-laboratory clinical sleep recording systems over-filter airflow signals well beyond AASM criteria: High pass filters (baseline drift filter) should be set to “off” (per AASM recommended filter settings); while the AASM recommended filter setting criteria allows up to 0.03 Hz, even this level compromises advanced flow waveform analysis. Low pass filters, often used by technicians to remove snoring vibrations to assess score flow reductions for hypopneas, should be no lower than 12.5 Hz to evaluate flow limitation (Mann et al., 2019, 2021), but AASM recommends no lower than 100 Hz (equivalent to “off” for sampling rates 25–200 Hz). The field is also lacking a standardized system for signals storage (e.g., signal labels vary widely) and annotations tabulations (e.g., event and epoch names vary widely, often durations are not available) to facilitate automated analyses. Manual sleep and event scoring will presumably be replaced with automatic scoring, that for many years may require manual quality control review. The software for analyzing traits needs to be at the fingertips of clinical sleep laboratories through data uploading or built as an add-on to commercially available sleep systems so that summary data can make their way into the “future PSG report.” The feasibility of this has been demonstrated through the PUPpy cloud-based implementation of the method (Finnsson et al., 2021). Clinicians will need guidance on how to interpret trait data with respect to the reliability and the likely responses to different therapies. For this to be evidence-based, substantially larger datasets containing raw PSG data before and after different therapies are needed to better define the expected treatment responses for different endotypic subgroups, ultimately allowing a clinician to see the expected treatment effects (and 95% confidence) for a host of therapies based on their values of collapsibility, loop gain, etc. Such data is also needed to better define “high” and “low” values for a given trait. In the meantime, the current use of endotypic traits in recent clinical trials is encouraging. Subsequently, larger studies will be needed to show that knowing the endotypic traits provides better patient outcomes and is more cost-effective than not knowing them (and using trial-and-error to select therapeutic interventions). Potentially, these studies could extend to whether endotypes should serve as an aid in deciding if a patient's OSA is severe enough to warrant treatment or not, although novel phenotypic traits such as hypoxic burden (Azarbarzin et al., 2019), heart rate response to events (Azarbarzin et al., 2021), or baseline levels of sleepiness/hypertension (Randerath W. J. et al., 2021) could be better suited for that purpose. There is a substantial amount of challenging work to do for investigators, clinicians, and engineers to make precision sleep medicine a reality.

In summary, it is now attainable to estimate individual differences in the key traits contributing to sleep apnea–collapsibility, compensation, arousal threshold, and loop gain–through analysis of ventilation and ventilatory drive in a routine clinical sleep study, i.e., without invasive measurements or specialized operators. Multiple challenges are being overcome for the translation of these endotypic traits into clinical practice. We consider that such mechanistic information will facilitate precision medicine for OSA, and in doing so, make clinical sleep medicine a more enriched and rewarding field.

## Ethics statement

This study involved secondary analysis of anonymized data. MESA and MrOS were multi-site cohort studies; data is publicly available at the National Sleep Research Resource Repository, at www.sleepdata.org/datasets and https://mrosonline.ucsf.edu/ respectively. The studies were reviewed by Institutional Review Boards at each site. The analysis of data from the CMUH cohort was reviewed and approved by the Institutional Review Board of China Medical University Hospital (CMUH109-REC3-018). All participants for all studies provided written informed consent.

## Author contributions

EF, EA, SH, JÁ, and SS contributed to the conception of the review. EF, EA, SH, and JÁ wrote sections of the manuscript with revisions from SS. SS, W-JC, RA, and L-WH contributed the data. ÞS and RA organized the data and performed statistical analysis. EF performed statistical analysis and created all figures. JÁ and SS supervised the work. All authors read, revised, and approved the submitted manuscript.
